# Developing a fluorometric urease activity microplate assay suitable for automated microbioreactor experiments

**DOI:** 10.3389/fbioe.2022.936759

**Published:** 2022-09-14

**Authors:** Frédéric M. Lapierre, Isabel Bolz, Jochen Büchs, Robert Huber

**Affiliations:** ^1^ Munich University of Applied Sciences HM, Munich, Germany; ^2^ Chair of Biochemical Engineering (AVT.BioVT), RWTH Aachen University, Aachen, Germany

**Keywords:** urease, enzymatic assay, MICP, microbioreactor, automation, sporosarcina pasteurii

## Abstract

Quantifying urease activity is an important task for Microbial Induced Calcite Precipitation research. A new urease activity microplate assay using a fluorescent pH indicator is presented. The method is also suitable for automated measurements during microbioreactor experiments. The assay reagent consists of the green fluorescent pH-indicator fluorescein, urea and a phosphate buffer. After sample addition, the microbial urease hydrolyses urea, which results in a pH and hence fluorescence increase. The fluorescence signal can be measured with a microplate reader or with the microbioreactor system BioLector, allowing for automated urease activity measurements during cultivation experiments. In both measurement systems, the fluorescence signal slope highly correlates with the urease activity measured offline with standard methods. Automated measurement is possible, as no sample preparation such as centrifugation or adjusting of the optical density is required. The assay was developed so that the culture samples turbidity, salinity or buffer concentration does not have a negative impact on the fluorescence signal. The assay allows for straightforward, non-hazardous, parallelized, cheap and reliable measurements, making research on ureolytic bacteria for Microbial Induced Calcite Precipitation more efficient. The assay could be adapted to other enzymes, which have a strong impact on the pH value.

## 1 Introduction

Urease (urea amidohydrolase, EC 3.5.1.5) is naturally found in plants, algae, fungi and bacteria (Hausinger, 1987). The nickel-containing enzyme catalyses the hydrolysis of urea to ammonia and carbamate (Kappaun et al., 2018). Carbamate spontaneously decomposes to an additional ammonia molecule and carbonic acid, resulting in an overall pH increase in aqueous solution (Mazzei et al., 2020). The overall reaction can be summarized by the following chemical equation:

$$CH_{4}N_{2}O+3H_{2}O\to 2NH_{4}^{+}+HCO_{3}^{-}+OH^{-}$$ (1)

Ureolytic bacteria are used for microbial induced calcite precipitation (MICP). The increased alkalinity and the presence of dissolved CO_2_ resulting from microbial urease activity enable the precipitation of hardly soluble calcite, when calcium ions are added. This process is considered for multiple applications, such as metal and radionuclide remediation, CO_2_ sequestration, restoration on construction materials (Zhu and Dittrich, 2016) or dust control (Meyer et al., 2011). Urease activity is considered to be a key factor for MICP performance (Konstantinou et al., 2021). Multiple studies use different techniques to increase the urease activity of a bacterial culture (Achal et al., 2009; Omoregie et al., 2017; Zhao et al., 2019), or isolate new ureolytic microorganisms (Li et al., 2013; Krishnapriya et al., 2015; Kim et al., 2016; Zhao et al., 2017). Quantification of urease activity plays an important role for MICP research and application (Cui et al., 2022). Besides this, detection of urease activity is also relevant in agriculture as well as in medicine (Mazzei et al., 2020). However, measuring urease activity can be considered time consuming and laborious. No high-throughput assay allowing easy quantification of urease activity for many samples in parallel without extensive sample preparation has been published yet.

At present, the urease activity of one sample can be determined by different direct or indirect detection methods. Commonly, colorimetric tests as the Berthelot or Nessler assay are used (Vermelho, 2013). The Berthelot assay uses phenol and the Nessler assay uses potassium tetraiodomercurate for detection of ammonia production over a certain time period in order to calculate the urease activity. Both reagents are highly toxic and dangerous to the environment, making them undesirable for everyday laboratory work. Phenol as a reagent of the Berthelot test can be substituted with the less harmful phenols as sodium salicylate or 2-phenylphenol (Rhine et al., 1998), making an adapted assay more applicable. Nevertheless, both colorimetric tests consist of numerous timed pipetting steps (Vermelho, 2013), making these tests prone to errors and difficult to parallelize and automate. Alternatively, the indirect conductivity assay first published by Chin and Kroontje (1962) and later established for MICP-relevant microorganisms by Whiffin (2004) is often used as well. The conductivity assay is easy to perform. Here, an urease-positive sample is added to an urea solution, resulting in the formation of ions. The conductivity change over three up to 8 min is monitored. The slope of the conductivity signal correlates with the urease activity (Whiffin, 2004). In our experience, due to the experimental setup using conductivity probes, the test requires at least 0.5 ml of sample. This makes this test not suitable for microbioreactor experiments with cultivation volumes of 1 ml or less, without spending one entire culture. In addition, to the best of our knowledge, there are no high throughput conductivity measurement systems commercially available, which would allow for parallelized or even automated assay procedures. As the determination of enzymatic activity, a time dependent parameter, inherently takes several minutes, measuring one sample after another with any method does add up and can consequently be considered time consuming. Other methods as the quantification of ^14^CO_2_ by scintillation counting after hydrolysis of radiolabeled urea or titration are posing the same issues, and can also be considered time consuming and laborious, and require specific laboratory instruments (Vermelho, 2013).

One promising assay for parallelized urease activity quantification using a microplate reader is described by Onal Okyay and Frigi Rodrigues (2013). This test is based on quantifying a pH shift resulting from the urease activity. Measuring the pH directly via probes to quantify urease activity was already published by Bibby and Hukins (1992). Contrary to the test by Bibby and Hukins, the assay from Onal Okyay and Frigi Roridgues does not measure the pH with probes, but is based on a color-changing medium commonly labelled as Stuart Broth, which was originally used as a method for classification of bacteria into ureolytic and non-ureolytic microorganisms (Rustigian and Stuart, 1941). The medium consists of the pH indicator phenol red, urea and a phosphate buffer. During this assay, an ureolytic microorganism hydrolyses urea, which results in a pH increase, which is made visible by a color shift of the pH indicator from yellow to red. Non-ureolytic microorganism do not hydrolyse urea, and consequently, no color shift can be observed. This color shift or its absence allows for easy distinction between ureolytic and non-ureolytic microorganisms. Similar tests for visualization of enzyme-catalyzed reactions using pH indicators have been published (Moris-Varas et al., 1999; Reymond, 2006; Rachinskiy et al., 2009).

**FIGURE 1:**
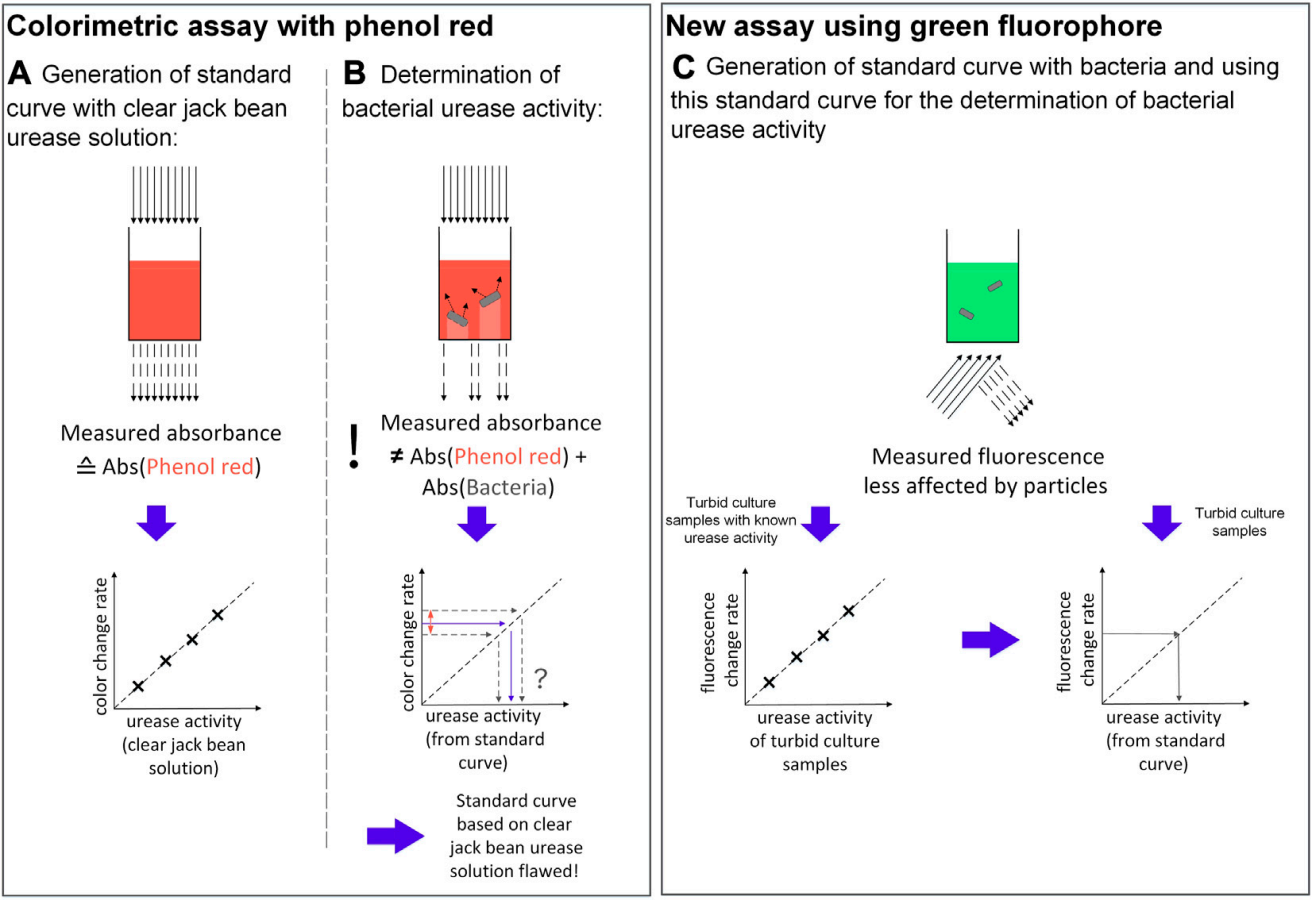
Comparison of the colorimetric assay by Onal Okyay and Frigi Rodrigues (2013) illustrating one well of a 96-well microplate for **(A)** urease solutions and **(B)** bacterial suspensions, as well as **(C)** the proposed fluorometric assay in this study, using sodium fluorescein in a 48-well plate.

In the study of Onal Okyay and Frigi Rodrigues (2013), the color change of 270 *μ*L Stuart Broth with 30 *μ*L sample (resulting in an 10:1 ratio) was monitored every minute over an hour by spectrophotometry in a shaken 96 well plate at 560 nm, which corresponds to one of the two absorbance maxima of phenol red. In order to generate a standard curve, the change in slope was correlated with the known urease activity of jack bean urease (Figure 1A). This correlation was used to determine the urease activity of nine different microorganisms (Onal Okyay and Frigi Rodrigues, 2013) (Figure 1B). However, while this method allows for easy parallel kinetic measurement of absorbance and can therefore be considered as high-throughput, the method requires extensive sample preparation prior to that. First, the bacterial samples have to be washed with phosphate-buffered saline (PBS) and adjusted to an optical density (OD) at 600 nm of 0.5, before addition to the Stuart Broth in the microplate. These preparation steps therefore require centrifugation, cell washing and extra OD measurements, taking additional time and effort, making the assay again as time consuming and laborious as non-high-throughput assays. Also, adjusting sample OD is an extra step prone to errors. The same is true for similar high throughput assays, which are making use of color shifting pH indicators as the assay recently published by Cui et al. (2022).

The study above does not explicitly explain the reason for the sample preparation (Onal Okyay and Frigi Rodrigues, 2013). Based on our preliminary experiments (see Supplementary File 1), the reason for washing the culture is to avoid pH buffering effects from the culture medium, which have a decreasing effect on the color shift rate of the pH indicator, resulting in an underdetermined enzyme activity. Adjusting the OD600 to 0.5 is necessary as the bacterial suspension also absorbs light at 560 nm, the absorbance wavelength of the assay. Using samples with different OD600 therefore results in a positive shift of the absorbance curves, again having an impact on the determined urease activity (see Supplementary File 1). However, the bacteria do not only absorb light, but also scatter it (Stevenson et al., 2016) (Figure 1B). Consequently, the apparent measured absorbance does not correspond to the sum of the absorbance of the bacteria plus the absorbance of the pH indicator (Mayerhöfer et al., 2019), making a standard curve based on a clear jack bean urease solution flawed when measuring turbid samples. All in all, while the assay is based on the right approach, some adjustments have to be made in order to efficiently measure urease activity of multiple bacterial samples for MICP research.

Here, we propose an adapted high-throughput urease activity microplate assay, which does not require any sample preparation. The assay works with every microplate reader capable of kinetic fluorescence measurements. Additionally, the assay is also suitable for automated microbioreactor experiments. While high-throughput microbioreactors enable fast bioprocess optimization by online monitoring of biomass, fluorescence, pH or dissolved oxygen of many samples in parallel, they do not allow for enzyme activity measurements yet. However, periodic measurement of enzyme activity is crucial for some bioprocess developments, for example for MICP research. The here established assay closes this gap. To the best of our knowledge, this is the first assay allowing for enzyme activity measurement of culture samples during microbioreactor cultivation itself, without requiring additional measuring devices. This makes determination of external influences on microbial urease activity in different growth phases much simpler.

Among other adaptations, this improved assay is mainly realised by substituting the phenol red of the Stuart Broth with the fluorescent pH indicator sodium fluorescein (Figure 1C). Compared to colorimetric assays, a fluorometric assay is generally less affected by particles like bacteria, as these particles should not show the same fluorescent properties as the fluorophore (Kensy et al., 2009). Consequently, emission detection is assumed to be less prone to errors from particle light absorption, scattering or shading. Fluorescein and its derivates are used as pH probes for manifold purposes, such as for pH monitoring of bacterial growth or fluorescent titration (Le Guern et al., 2020). In a previous study by Suye et al. (1999), sodium fluorescein was already successfully applied in order to detect urea by addition of urease. Similarly, Chaudhari et al. (2017) developed a biosensor consisting of a fluorescein-derived fluorophore co-immobilized with urease in alginate for estimating urea and pH of clinical samples. In comparison to mentioned studies, the parameters are switched for the assay presented in this study; this means, urea and sodium fluorescein are used to determine the urease activity.

To begin with, the absorbance spectrum and emission curve of sodium fluorescein at different pH values are determined in order to develop a standard protocol. Then, *Sporosarcina pasteurii*, the bacterium most-often used in MICP research (Ma et al., 2020), is cultivated in a fully automated microbioreactor experiment, while its urease activity is regularly monitored during cultivation. Finally, the general applicability of the fluorometric assay for different culture media compositions and OD values of the samples is demonstrated for measurements with the microbioreactor system as well as with a microplate reader.

## 2 Methods

### 2.1 Development of the fluorescein assay protocol

**FIGURE 2:**
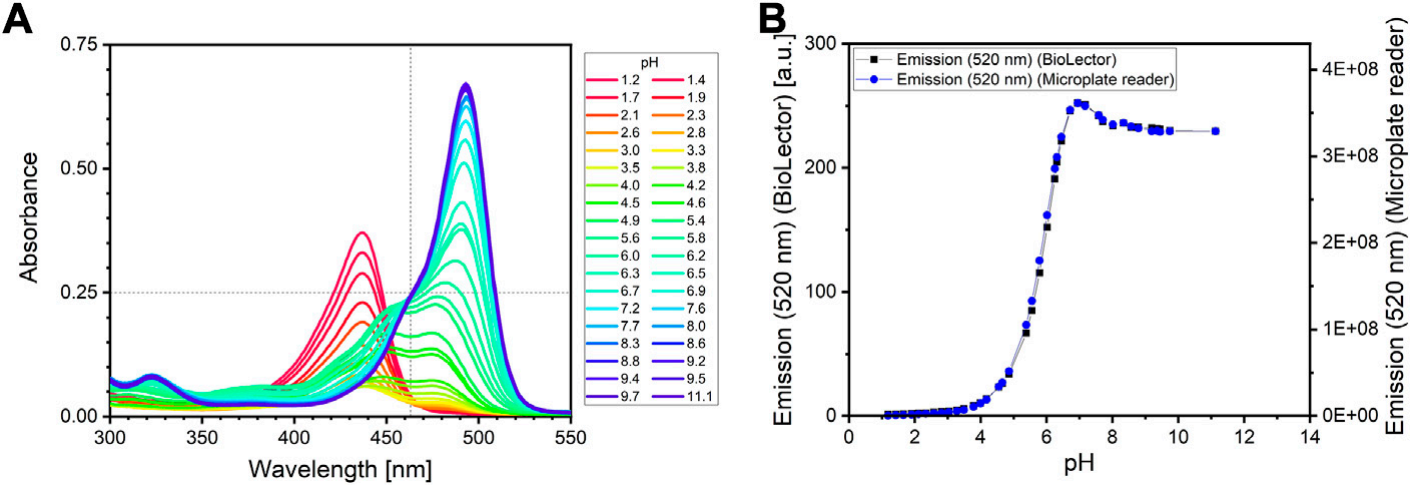
**(A)** Absorbance spectrum of a sodium fluorescein solution at different pH. The isosbestic point was found at 463 nm (indicated be the dotted vertical line). The fluorescein reagent is adjusted to an absorbance at wavelength 463 nm of 0.25 (indicated by the dotted horizontal line). Measurement of 200 *μ*L fluorescein solution in a 96 well microplate (*μ*clear, Greiner BioOne, Kremsmünster) using a microplate reader (SpectraMax iD3, Molecular Devices, United States). **(B)** Emission intensity of sodium fluorescein at 520 nm after excitation at 488 nm at different pH. Measurement of 1,000 *μ*L fluorescein solution in a 48 well microplate (Flower Plate, Beckman Coulter Life Sciences, Baesweiler) using the BioLector I (Beckman Coulter Life Sciences, Baesweiler) (left axis), or 200 *μ*L fluorescein solution in 96 well microplate (*μ*clear, Greiner BioOne, Kremsmünster) using a microplate reader (SpectraMax iD3, Molecular Devices, United States) (right axis) (mean values, N = 2). The barely visible error bars depict the standard deviation.

Here, the three development steps of the assay protocol development are described. In order to guarantee reliable sample analysis, the fluorescent assay reagent must have the exact same composition for every experiment. An equivalent concentration of the sodium fluorescein for every application of the assay is especially important to ensure the same absolute fluorescence values. However, weighing very small amounts (<5 mg) of the fluorescent chemical on a scale may result in variations of the measured fluorescence signal due to instrumental (Druzdzel and Wieneke, 2016) or human inaccuracy. A more reliable way to prepare the exact same fluorescein reagent every time was found by making use of the isosbestic point of the absorbance spectrum of fluorescein. The isosbestic point is defined as the wavelength, at which the absorbance of a solution remains constant at different pH values. As can be seen in Figure 2A, the isosbestic point is at 463 nm, but only at pH values above 6.5. This can be explained by the multiple ionic forms of fluorescein; the single isosbestic point at pH values above 6.5 is a result from the superposition of the absorption spectra of the di-anionic and mono-anionic form of fluorescein (Le Guern et al., 2020). More details on this are provided in Supplementary File 2. For the fluorescein reagent preparation, a concentrated alkaline sodium fluorescein stock solution is diluted with water to a defined absorbance (0.25) at the isosbestic point (463 nm).

The choice of the buffer system and the total buffer concentration of the fluorescein reagent as well as the ideal sample-to-reagent ratio were determined in a preliminary experiment (see Supplementary File 3). After mixing the fluorescein solution with the phosphate buffer and urea, the reagent is then adjusted to a pH of 6. No considerable impact on the urease activity is expected at pH 6 (Whiffin, 2004), but a strong change in the fluorescence signal is expected with increasing pH, as can be seen in the pH-dependent emission curve of fluorescein illustrated in Figure 2B.

A sample-to-reagent ratio of 1:20 was found to be suitable for the analysis (see Supplementary File 3). Therefore, 50 *μ*L of each sample is pipetted to a designated well of a multiwell plate. Afterwards, 950 *μ*L of the fluorescein reagent is added to each sample. The kinetic change of the fluorescence signal can be either detected with a microplate reader or directly with the microbioreactor system (Figure 2B). The measurement results of both devices are highly correlated (see Supplementary File 4). The option to measure green fluorescence in the BioLector is originally installed to detect green fluorescent protein (GFP) as a reporter of protein expression. Please note, that the standard BioLector (as it is sold by the manufacturer) is not able to generally measure fluorescence, but only green fluorescence at the here defined wavelengths. Fluorescence at other wavelengths can only be measured after installing additional filter modules. Consequently, fluorescein was chosen as the pH indicator, as it has similar excitation and emission wavelengths as GFP. The BioLector is also able to online monitor pH directly, when specific microplates with optodes are used. However, pH measurement is only reliable at a range typical for microbial cultures (between pH 4 and 7.5, depending on the microwell plates). This range is not suitable for accurate urease activity measurement, as this result in an alkaline pH (>9). Measuring fluorescence directly in the microbioreactor makes use of the automated pipetting platform and allows for direct measurements of a culture sample, enabling fast and effortless bioprocess optimization. In this case, 50 *μ*L of a culture sample from a culture well is pipetted via the pipetting robot to 950 *μ*L fluorescein reagent in an measuring well. In order to avoid extensive photobleaching from excitation by the measurement system itself, the reagent is only pipetted to a measuring well before addition of the sample. The resulting fluorescence signal after the addition of the sample is at the beginning increasing linearly due to the increasing pH from ureolysis (Figure 4B). After some time, the slope decreases and the signal reaches a plateau. The urease activity correlates with the linear portion of the fluorescence signal.

Maintaining a constant temperature (here: 30°C) is crucial for this assay, as the enzymatic reaction itself is temperature dependent (Whiffin, 2004), and the fluorescence intensity of fluorescein also depends on its temperature (Mishra et al., 2016). Practically, this was no issue, as both measurement systems, the microbioreactor and the microplate reader, include temperature regulation. Using cold fluorescein reagent directly from the laboratory refrigerator resulted in nearly the same signal as using reagent at room temperature, as the reagent is acclimated very fast during measurement (see Supplementary File 5).

### 2.2 Assay protocol

**Fluorescein reagent preparation.** For the fluorescein reagent preparation, a concentrated alkaline sodium fluorescein stock solution is prepared first. This stock solution contains 100 mg/L sodium fluorescein (AppliChem, Darmstadt) and 500 mg/L NaOH pellets. The stock solution is then diluted to an absorbance (96-well microplate, 200 *μ*L filling volume) at the isosbestic point (463 nm) of 0.25 (±2%). This diluted solution is then added to pre-weighed dry phosphate buffer (final concentration: 5.304 g/L K_2_HPO_4_, 51.273 g/L KH_2_PO_4_, resulting in a total buffer concentration of 0.4 M) and urea (final concentration: 30 g/L). After mixing, the reagent is then adjusted to a pH of six using solid NaOH pellets in order to avoid any dilution. The fluorescein reagent solution was freshly made before each experiment. In order to avoid photobleaching by exposure with ambient light, aluminium foil was wrapped around falcon tubes or other storage vessels holding the reagent.

**Microorganism and pre-cultivation.** *Sporosarcina pasteurii* (DSM33) was obtained from the German Collection of Microorganisms and Cell Culture GmbH (DSMZ, Braunschweig). For precultivation and main cultivation experiments, *S. pasteurii* was cultivated in CaSo^+^ medium, derived from previously published findings (Lapierre et al., 2020). CaSo^+^ medium contains 10 g/L glucose, 20 g/L urea, 1.7 g/L K_2_HPO_4_, 15 g/L peptone from casein, 5 g/L peptone from soy, 5 g/L NaCl, 50 ml/L micronutrient stock solution and 5 ml/L iron stock solution. The micronutrient stock solution consists of 8.54 g/L MgCl_2_⋅ 6H_2_O, 0.56 g/L MnSO_4_⋅ 3H_2_O, 0.18 g/L ZnSO_4_⋅ 7H_2_O, 0.085 g/L CoSO_4_⋅ 7H_2_O, 0.08 g/L CuSO_4_⋅ 5H_2_O, 0.06 g/L (NH_4_)_6_Mo_7_O_24_⋅ 4H_2_O, 0.2 g/L NiCl_2_⋅ 6H_2_O and 0.2 g/L EDTA. The iron stock solution consists of 1 g/L FeCl_3_⋅ 6H_2_O and 1 g/L FeCl_2_. The medium was adjusted to a pH of 7.5 with NaOH when necessary and sterile filtered afterwards. Precultivation was carried out via cultivation at 30 °C using 250 ml shake flasks with a filling volume of 25 ml at a shaking frequency of 300 rpm and a shaking diameter of 50 mm in a darkened incubator shaker (LT-X, Kuhner AG, Birsfelden). Cell growth during shake flask cultivation was monitored using a backscatter measurement system (Cell Growth Quantifier, CGQ, aquila biolabs, Baesweiler). 

**FIGURE 3:**
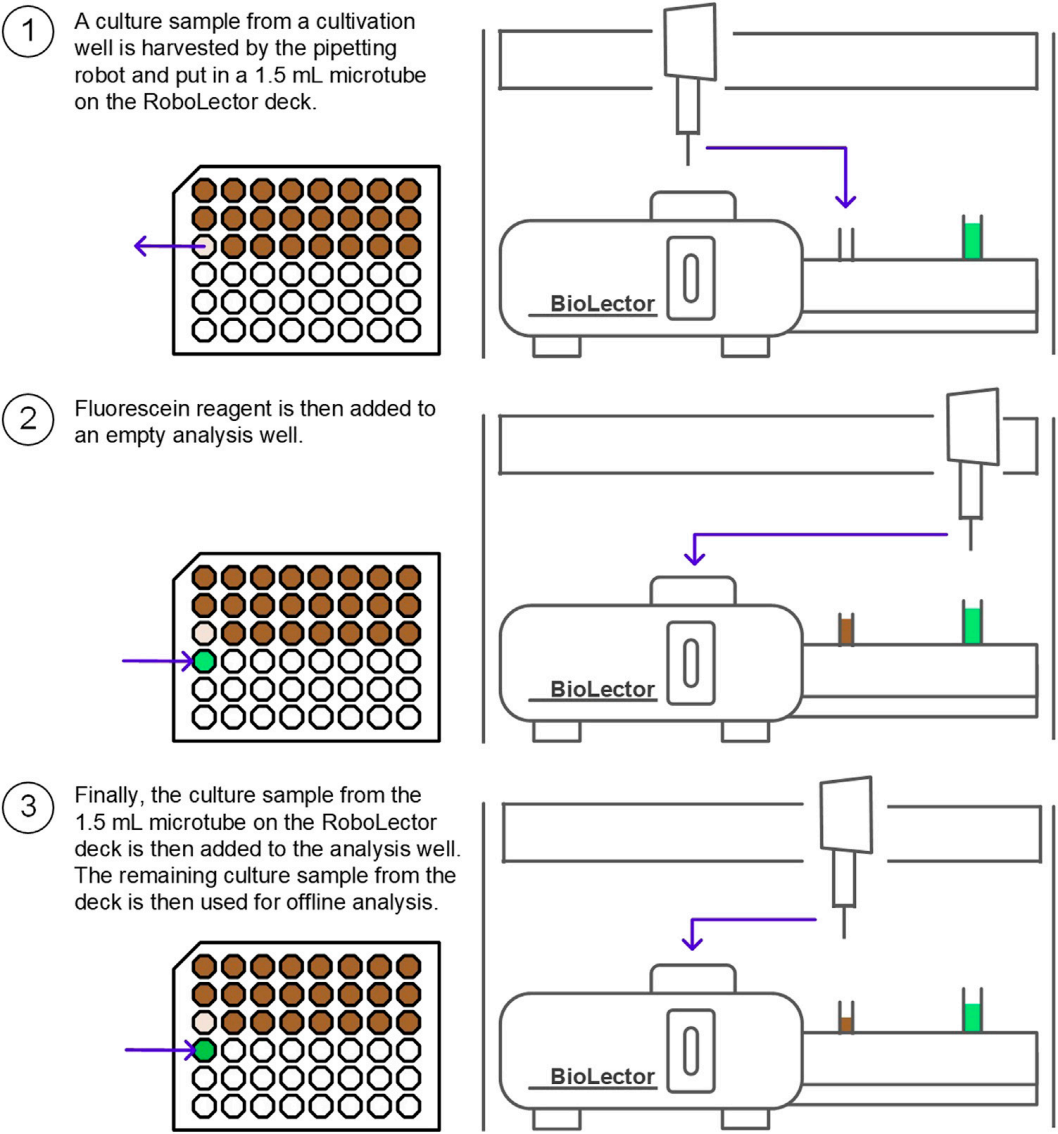
Overview of urease activity assay for automated microbioreactor experiments. A culture sample is illustrated in brown and the fluorescein reagent is illustrated in green. On the left, a top view of the microplate is shown. On the right, a frontal view of the RoboLector system is illustrated. When no offline analysis is necessary, 70 *μ*L of culture are enough for sample analysis.

**Microbioreactor cultivation and fluorescence measurement**. An overview of the procedure is illustrated in Figure 3. The BioLector I microbioreactor system combined with the RoboLector L pipetting robot for automated fermentation (Beckman Coulter Life Sciences, Baesweiler) was used under a laminar flow work bench (LAMINO Workplace L-18–10-T, Asys, Dornstadt). Bacterial growth in 48-well baffled microplates (Flower Plates MTP-48-B, Beckman Coulter Life Sciences, Baesweiler) was monitored via backscatter quantification at 620 nm every 5 min. Cultivation was carried out at 30 °C with a shaking diameter of 3 mm, a shaking frequency of 1,200 rpm and a filling volume of 1,200 *μ*L. The humidity control of the BioLector I system was activated (resulting in relative humidity ≥85%) in order to reduce evaporation of the culture medium. The detector sensitivity for cell growth monitoring was set to a gain of 20 in the software. For the fluorescence measurements, the GFP detector sensitivity was set to 30. For the automated urease activity experiment, 1,100 *μ*L sample were harvested by the pipetting robot and put in a 1.5 ml microtube on the RoboLector deck. 950 *μ*L fluorescein reagent, stored in a light-protected falcon tube on the RoboLector deck, were then added by the robot to an empty analysis well. Finally, 50 *μ*L culture sample from the RoboLector deck are then added to the analysis well. The remaining culture sample on the RoboLector deck was then used for the conductivity measurement. When no offline analysis is necessary, 70 *μ*L sample are sufficient for urease activity analysis, which corresponds to the sum of the sample volume necessary for the assay (50 *μ*L) and the dead volume remaining at the vessel bottom (20 *μ*L, specified by the manufacturer). The RoboLector is also able to transfer samples from a culture well directly to a sample well. However, this was found to be less precise than transferring the sample to the RoboLector deck first (data not shown). 

**Spectrophotometric measurements**. The spectrophotometric and fluorescence measurements were carried out using a microplate reader (SpectraMax iD3, Molecular Devices, United States). For fluorescein spectrum analysis, fluorescein reagent preparation and OD600 determination, 96 well microplates (*μ*clear, Greiner Bio-One, Kremsmünster) with a filling volume of 200 *μ*L were used. For kinetic fluorescence measurements, 50 *μ*L culture sample and 950 *μ*L fluorescein reagent were added to a 24 multiwell plate (Cellstar 24 Well Cell Culture Plate sterile, greiner bio-one, Frickenhausen) before sealing and measured at 520 nm after excitation at 488 nm. The excitation and measurement wavelengths were adapted from the BioLector system, which is able to detect green fluorescence only at these wavelengths. This allowed for easy comparison of both measurement systems. A 24 multiwell plate was used, as it could hold 1 ml per well, allowing for easy comparison to 48 well measurements using the BioLector. Deionized water was used as a blank. The temperature was set to 30°C. The plate was monitored every 5 min. Before and between reads, the multiwell plate was shaken by the microplate reader on double orbital shake mode with low shaking intensity (corresponds 448 rpm, specified by the manufacturer). The measurement was stopped after a plateau of the measuring signal was reached.

**Microplate sealing.** For the here established assay, using a gas-tight sealing foil is important. Sigurdarson et al. (2020) noted ammonia volatilization, when using the phenol red urease activity microplate assay, resulting in an unwanted color shift of neighbouring wells. Similar unwanted pH shifting effects by ammonia volatilization were expected here. Therefore, the 24-well multiwell plate was sealed using adhesive sealer (EASYseal, Greiner Bio-One, Kremsmünster). Sealing the 48-well plate of the automated microbioreactor measurement was a challenge, as the gas-tight sealing foil also has to be pierceable by the RoboLector pipetting robot. Here, a solution was found by first sealing the entire 48-well plate with an adhesive gas-permeable foil and adding a silicon layer on top, as suggested by the manufacturer (Sealing Foil F-GPRS48-10, Beckman Coulter Life Sciences, Baesweiler). The silicon layer has ventilating holes, allowing for sufficient gas transfer for aerobic cultures. These ventilating holes are then again sealed with an adhesive foam foil (HJ-Bioanalytik, Erkelenz), but only at wells determined for fluorescence measurements, preventing any negative effects from ammonia volatilization.

**FIGURE 4:**
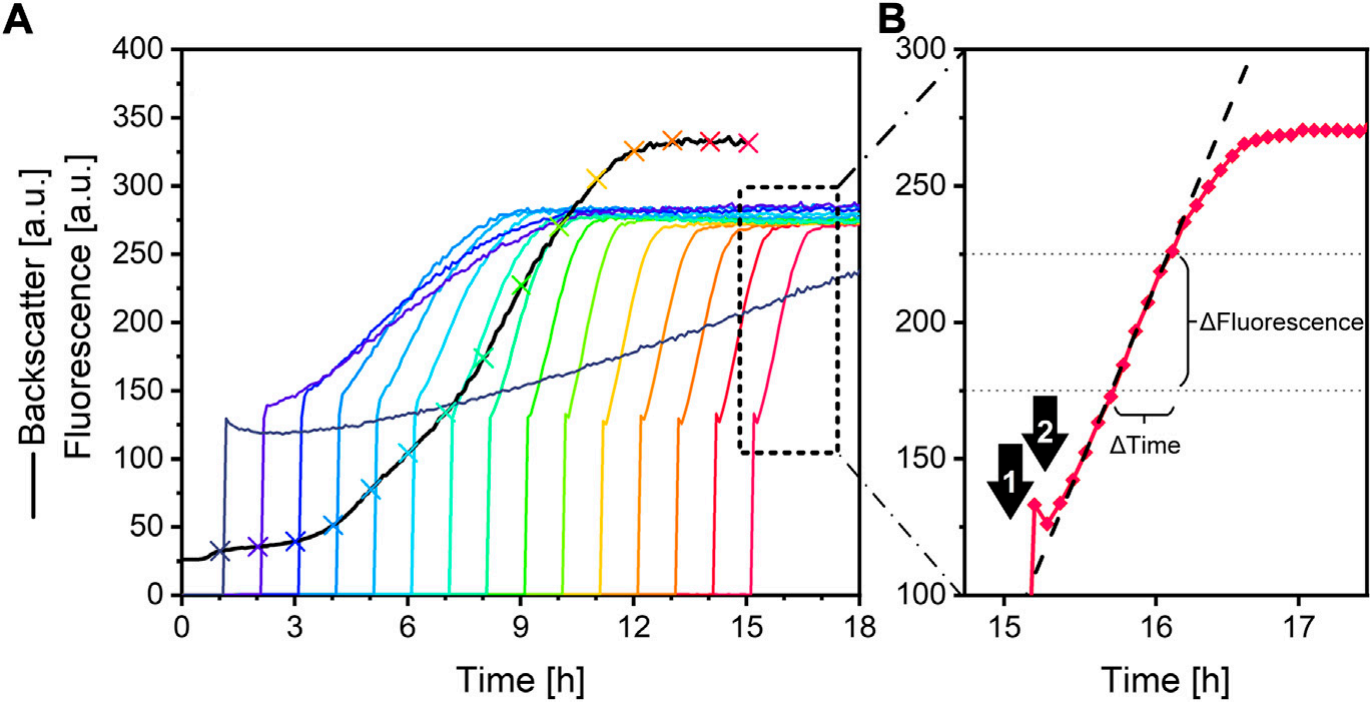
**(A)** Backscatter (black line) and fluorescence data (multicoloured lines) from a microplate cultivation of *S. pasteurii* with automated sampling and urease activity measurement. The crosses, coloured to their according fluorescence signal, indicate culture sampling. **(B)** Fluorescence curve (red diamonds) after sample addition for urease activity measurement. The arrows are indicating the measurement points (1) after the fluorescein reagent is added to the well, and (2) after the ureolytic sample is added to well. The black dashed line indicates the linear fit of the signal between 175 a. u. and 225 a. u. Culture conditions: 48-well Flower Well Plate, culture filling volume 1,200 *μ*L, reagent filling volume 1,000 *μ*L, shaking frequency 1,200 rpm, shaking diameter 3 mm and temperature 30 °C.

**Fluorescence signal interpretation.** As illustrated in Figure 4B, the linear portion of the fluorescence signal is used for urease activity determination. The slope, with the unit a. u./h, was determined with the corresponding SLOPE function in Excel 2016. For very low urease activity samples, a photobleaching effect resulted in a signal decrease before the signal increases at a later point (Figure 4A, purple line). For this reason, only data points between 175 a. u. and 225 a. u. were used for data interpretation.

**pH measurements.** For pH measurements and adjustments, a portable pH meter (Seven2go pH Meter, Mettler Toledo, Giessen) with a micro probe (pH Electrode InLab Micro, Mettler Toledo, Giessen) was used.

**Determination of urease activity *via* conductivity assay.** Three conductivity electrodes (InLab 751, Mettler Toledo, Giessen) were inserted in 24 ml of an 1.1 mol/L urea solution. After addition of 0.5–1 ml of bacteria culture and filling up to 25 ml with the urea solution when necessary, measurement was started and carried out with constant stirring with a magnetic stirrer for 5 min. The average slope of the mean of the three linear conductivity signals was calculated and correlated with the urease activity. The assay was performed at room temperature.

**Determination of urease activity *via* Berthelot assay.** Urease activity was determined via the Berthelot method in order to create a standard curve for the conductivity assay (Vermelho, 2013). For ammonium detection, a commercial cuvette test was used (LCK 502 Ammonium-Nitrogen, Hach Lange, Loveland). The absorbance was measured and automatically converted to ammonium concentration values by the spectrophotometer for water analysis (DR 3900, Hach Lange, Loveland). The cuvette test is designed for very high ammonium concentrations. Therefore, no dilution is required. Urease activity was determined by measuring the ammonium concentration increase simultaneously to the conductivity assay, here 15 min after addition of 1 ml sample to an urea solution. In order to calculate the ammonium increase, the initial ammonium concentration must be determined first. Therefore, 1 ml sample was added to 24 ml deionized water, before measuring the ammonium concentration. The absolute ammonium increase after 15 min calculates by subtracting the initial ammonium concentration from the ammonium concentration after addition to the urea solution, times the total sample volume, here 25 ml. The resulting value is divided by 15 min in order to determine the ammonium generation after 1 min. As one urea molecule is hydrolysed to two ammonium molecules (see Eq. 1), dividing the ammonium generation by two gives the urea consumption. In summary, the urease activity of a sample in enzyme units, defined as the amount of substrate hydrolysed in 1 minute, was calculated according to the following formula:

$$Urease\mathrm{activity}\left[U/mL\right]=\frac{\left(c{\left(NH_{4}^{+}\right)}_{t=15min}-c{\left(NH_{4}^{+}\right)}_{\mathrm{sampleinwater}}\right)\left[\mu mol/L\right]\cdot 0.025L}{15\min\cdot 1mL\cdot 2\frac{mol_{NH_{4}^{+}}}{mol_{\mathrm{Urea}}}}$$ (2)

## 3 Results

### 3.1 Microbioreactor experiment

**FIGURE 5:**
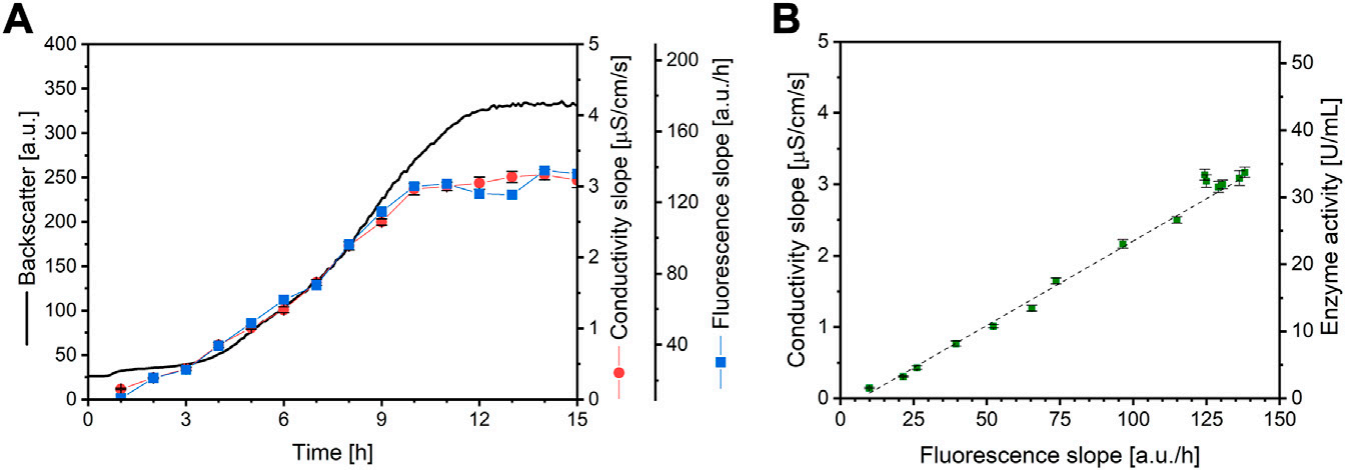
**(A)** Backscatter data (consistent line) from a microplate cultivation of *S. pasteurii* and urease activity data from standard conductivity measurements (red dots) as well as from the here established fluorescence assay (blue squares) **(B)** Standard curve resulting from the correlation of both methods (*R* ^2^ = 0.9914, N = 3). The error bars depict the standard deviation. As the correlation between conductivity slope and ammonium generation and therefore enzyme activity is known, the correlation between fluorescence slope and enzyme activity is also known and be calculated with the equation y = 0.2643 x - 2.2886.

In order to show applicability, a microbioreactor experiment is performed. In this case, urease-positive microorganism *Sporosarcina pasteurii*, the most often used microorganism for MICP (Ma et al., 2020), was cultivated using this system. A culture is sampled automatically by the pipetting robot every hour for 15 h. The sample was then used for the automatic fluorescence assay in the microbioreactor and for standard offline conductivity urease activity measurement. An overview of the resulting data obtained from the microbioreactor system can be seen in Figure 4A. The addition of fluorescein reagent to a well leads to a sharp signal increase starting from zero. After addition of the sample, the fluorescence signal drops slightly, which can be explained by sample dilution and photobleaching. After this, due to the ureolytic activity of the sample, the pH increases strongly, leading to a linear fluorescence signal. The signal plateaus after approximately 1 h, depending on the ureolytic activity of the sample. A higher urease activity results in a steeper increase of the fluorescence signal. An interpretation of the fluorescence signals is illustrated in Figure 5. As can be seen, the curve progression of the fluorescence assay matches the curve progression of the offline urease activity analysis by conductivity (Figure 5A). The fluorescence slope and the conductivity slope are highly correlated (Figure 5B). The measured urease activity progression also matches results published by other studies for *S. pasteurii* batch cultivation. Whiffin (2004) cultivated *S. pasteurii* ATCC 11859 in 20 g/L yeast extract medium with 20 g/L urea, and Ma et al. (2020) cultivated *S. pasteurii* BNCC 337394 in trypticase soy broth with 20 g/L urea. In both cases, the urease activity signals also plateaus earlier than the biomass signal, therefore before the culture entered stationary phase. The same observation was made here.

### 3.2 Influence of culture medium and OD600

One desired advantage of the here established assay is to make extensive sample preparation unnecessary. In the context of the urease activity analysis during a cultivation experiment, the assay should be independent of the medium composition and the optical density of the culture. As the assay is an optical method based on a pH shift, culture turbidity and medium buffer capacity were in particular taken into consideration. However, medium salinity was also tested. In order to test the influence of culture turbidity, medium buffer capacity and medium salinity, a culture sample and two dilutions of that sample (100%, 70%, 40%) were mixed with extra turbid medium, extra buffered medium or extra saline medium, respectively, before the fluorescence signal was monitored. Ideally, the determined urease activity should be independent of any additions. An increased OD600 or turbidity of a sample was simulated by addition of CaSo medium with urease negative inactive yeast (X-SEED Cell-FX, Ohly, Hamburg), resulting in equal OD600 for all three dilutions steps (see Supplementary 6). An elevated sample buffer concentration was simulated by addition of MOPS (3-(*N*-morpholino)propanesulfonic acid) buffer to the sample, resulting in an overall 0.2 M MOPS-buffer concentration of each sample. An increased sample salinity was simulated by the addition of extra NaCl to the sample, resulting in additional 2.5 g/L NaCl on top of the initial salinity of the samples.

**FIGURE 6:**
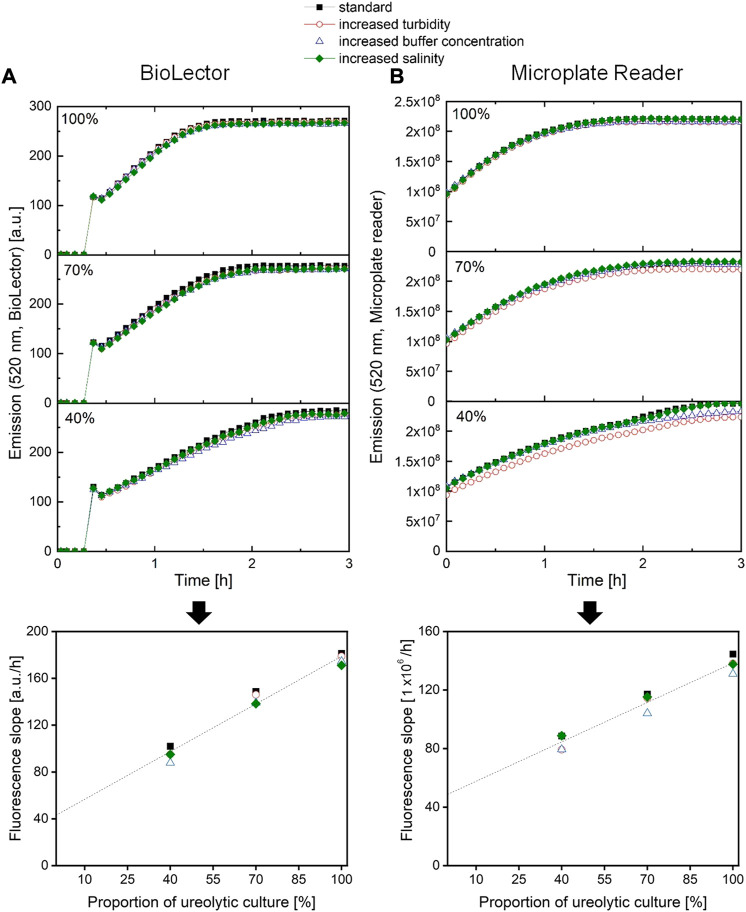
Fluorescence signals and their slope after sample addition for the same bacteria culture samples with different additives in order to increase sample turbidity, buffer concentration or salinity, **(A)** using a BioLector I (Beckman Coulter Life Sciences, Baesweiler) or **(B)** using a microplate reader (SpectraMax iD3, Molecular Devices, United States).

As can be seen in Figure 6, neither the increased OD600/turbidity, the increased buffer capacity, nor the increased salinity, had a strong impact on the fluorescence curve progression. Consequently, the fluorescence slope data indicating the urease activity do only slightly deviate from the standard sample without supplementations.

Additional cells, resulting in a higher OD600/turbidity, are not expected to interfere directly with the fluorescence measurement, as no relevant levels of biogenic green fluorescence can be detected, and the fluorescence of the reagent is at least around 5,000 times higher than the one from the bacterial culture (see Supplementary File 7). Nevertheless, additional cells or general turbidity (e.g. from soil samples) may absorb some of the excitation and emission light, resulting in a negative shift of the signal (Auld et al., 2004), as observed for microplate reader measurements (Figure 6B, 40%, red dots). Interestingly, this shift is not as obvious for the fluorescence data from the BioLector (Figure 6A). This can probably be explained by the different measurement conditions of both systems; the BioLector microbioreactor system is able to measure fluorescence, while the microplate is shaken at high speeds. This is not the case for the microplate reader. Here, the microplate can only be read after shaking has stopped, allowing for particles to sediment to the bottom of the well, partially blocking the light path, reducing the measured fluorescence intensity (Auld et al., 2004). In this case, the mean fluorescence signal slope is 6% lower compared to the unsupplemented standard sample for the microplate reader measurement, but only 3% lower on average when measuring with the BioLector system.

Increasing the medium salinity did not have any effects on the fluorescence measurement, and consequently, no effect on the fluorescence slope either. Contrary to this, a higher medium buffer concentration does result in slightly reduced fluorescence slopes (for example observed in Figure 6A, 40%, blue triangles). The fluorescence reagent consists of a phosphate buffer with a concentration of 0.4 M. Phosphate buffer and MOPS do have nearly the same pK_*a*_ of about 7.2. This allows for a rough calculation that a sample with extra MOPS buffer has a 2.6% increased total buffer concentration, slightly slowing down the pH shift and thus the fluorescence shift over time. In this experiment, the fluorescence slopes deviated even more than that, namely −10% for the microplate reader and −8% for the microbioreactor. In this case, it must be said that for measuring the urease activity of multiple samples with very different buffer concentrations, another determination method might be more accurate. However, besides that this scenario is rarely the found in practice, the standard conductivity method overestimated the urease activity of samples to an even higher degree compared to the standard samples, from 14% up to 41% (see Supplementary File 8). Overall, the assay can be considered accurate for most measurement scenarios, or at least more accurate than the standard conductivity method.

## 4 Discussion

As for most fluorescence assays, two limiting factors have to be considered: photobleaching and fluorescence quenching. Photobleaching is described as the permanent fluorescence loss after exposure of the fluorophore with excitation light (Harris and Mutz, 2006). In the context of the here established assay, three types of possible light exposure scenarios have to be considered:

• At first, exposure with ambient light of the fluorescein reagent during its storage must be avoided. For the experiments performed here, the reagent was at most 2 weeks old. The influence of the storage conditions on the reagent was not part of the scope of this study. No negative effects were detected here, similar to the findings by Harris and Mutz (2006), who describe that a fluorescein stock solution stored in the dark over a 7 month period exhibited no significant signal degradation.
• The second scenario, at which photobleaching might be a limiting factor, would be over the course of an automated microbioreactor experiment. This must be considered, as fluorescein reagent solution is stored in an open falcon tube on the RoboLector deck during the entire course of the experiment. Even though the solution can be protected from light *via* aluminium foil around the falcon tube to some degree, slight light exposure is expected. However, this does not have a notable photobleaching impact as can be seen in Figure 4. In this experiment, the initial fluorescence of the reagent without sample addition is measured every hour for 15 h. All initial fluorescence values are equal over the entire experiment. This indicates no fluorescence loss from photobleaching at this point.
• Lastly, starting with the measurements in the microbioreactor or the microplate reader, photobleaching of the reagent does occur. Fluorescence and backscatter measurements require light impulses to the measuring well, resulting in photobleaching. Photobleaching of fluorescein reagent without any sample addition is illustrated in Supplementary File 9. However, this issue can be considered negligible, as photobleaching occurs identical for every measurement well, making the measurement signals comparable to each other.

Fluorescence quenching is the reduction of fluorescence intensity of a fluorophore caused by the addition of another substances (Harris and Mutz, 2006). Quenching can be detected by creating a Stern–Volmer plot. No fluorescence quenching substance from the reagent assay or typical microbial samples was found. However, the Good’s buffer HEPES, widely used for microbial cultures, was found to be a fluorescence quenching substance (see Supplementary File 10). It is possible that a sample component might quench fluorescein and therefore negatively impacts the urease activity detection during this assay. However, interferences can occur with other colorimetric methods like the Berthelot method as well (Hach Company, 2019). When there is doubt, that a substance in a sample might quench fluorescein, it is recommended to create a Stern–Volmer plot. If the plot verifies fluorescence quenching, another urease activity assay would be advised. Nevertheless, it can be said that no big drawback by photobleaching or quenching is expected when using this assay.

In order to evaluate high throughput screening assays, the Z′-factor value established by Zhang et al. (1999) is often used. The Z′-Factor allows for comparison, optimization and validation of high throughput assays. An ideal assay has a Z′-factor of 1. Similar to Buß et al. (2016), the positive control was simulated by addition of 50 mg NaOH to 1 ml reagent, and plain reagent was used as the negative control. The resulting Z′-Factor is 0.94, which corresponds to “an excellent assay”, according to Zhang et al. (1999). The strictly standardized mean difference (SSMD) was found to be 24.7, which also classifies the assay as an “excellent assay” (Buß et al., 2016) (see Supplementary File 11 for both calculations).

The assay can also be considered cost-efficient. The reagent costs for the measurement of one sample are below 0.01 Euro. Urease measurement of 23 samples in a 24 multiwell plate results in total consumable costs of 2.10 Euro, mainly due to the cost for the multiwell plate itself. This is for example at least 85% cheaper than performing the Berthelot assay for ammonium detection in order to quantify urease activity (Vermelho, 2013), as this would cost 14.12 Euro for the same number of samples. Additionally, costs for disposal of toxic wastes have to be considered, when applying this method. That said, using the conductivity method by Whiffin, the same number of measurements would only cost 0.35 Euro, as only an urea solution is required and the beakers necessary can be cleaned and reused, making the conductivity assay even cheaper. However, using the conductivity assay includes a lot of idle time between individual measurements followed by pipetting steps, increasing overall costs due to human labour. All cost calculations can be found in Supplementary File 12.

Finally, the method presented here could be adapted for other turbid suspensions containing enzymes generating a shift of the pH value, such as amino acid decarboxylase, carbonic anhydrase, cholinesterase, hexokinase or ester hydrolysis by proteases (Reymond, 2006). In these cases, the substrate in the fluorescein reagent must be substituted with according substrates for the hydrolysis.

## 5 Conclusion

A new indirect urease activity microplate assay using a fluorescence pH indicator was developed. It was shown that no culture sample preparation is required at all, as neither culture turbidity, salinity or buffer concentrations notably affects the determined urease activity, making this test easy and straightforward to use. Consequently, the assay is also suitable for automation and was tested for an automated microbioreactor experiment. As the microplate assay allows for parallelized urease activity measurements, research on ureolytic bacteria e.g. for MICP can be more efficient. The high throughput assay was categorized as “an excellent assay” via determination of Z′-Factor and strictly standardized mean difference (SSMD). Additionally, the assay was found to be cheaper than the colorimetric Berthelot assay. Finally, the method can be adapted for other pH shifting enzyme reactions, which then also can be monitored by the BioLector microbioreactor system but also by a microplate reader as well.

## Data Availability

The raw data supporting the conclusion of this article will be made available by the authors, without undue reservation.
