# Supplementary material for: Developing a fluorometric urease activity microplate assay suitable for automated microbioreactor experiments
Source: Front Bioeng Biotechnol. 2022 Sep 14;10:936759. doi: 10.3389/fbioe.2022.936759 (PMC9515450; doi:10.3389/fbioe.2022.936759)
Supplement: Supplementary file 1 [file DataSheet1.PDF]

Supplementary Material

SUPPLEMENTARY FILE 1 – LIMITATIONS OF THE PHENOL RED ASSAY

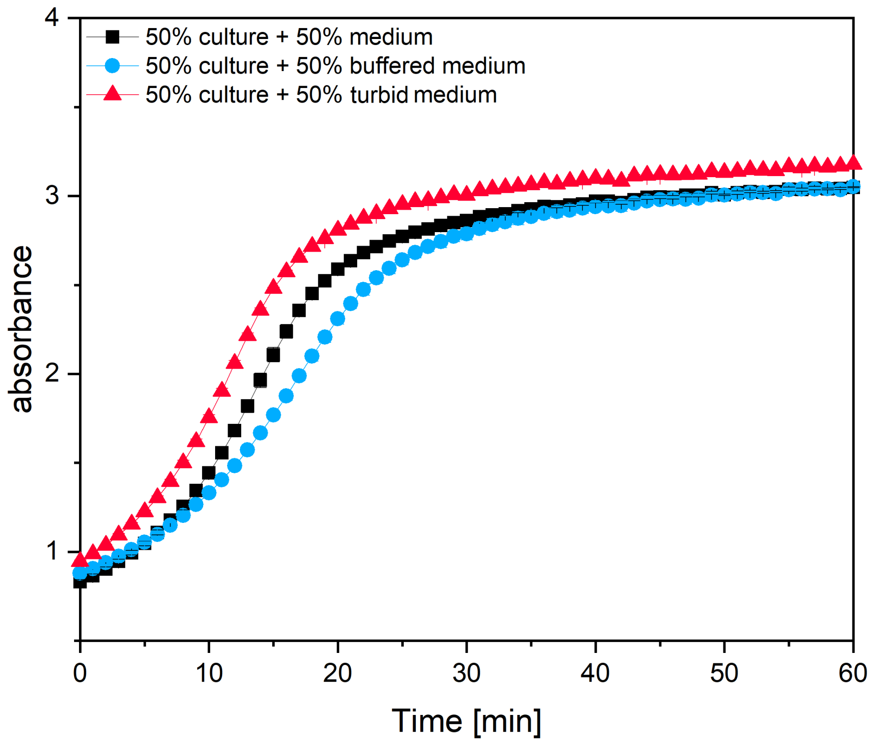

Figure S1. Absorbance curves of the same *S. pasteurii* culture sample diluted 1:2 with different media (in total each 20 μL) in Stuart Broth (180 μL) according to Onal Okyay and Frigi Rodrigues (2013). (mean value, N = 2 or 3). As in the original study, absorbance was measured every minute at 560 nm in a plate reader (SpectraMax iD3, Molecular Devices, USA) for 1 h at 28 °C in a slow shaking mode. Contrary to the original study, the microplate was sealed (EASYseal, Greiner Bio-One, Kremsmünster) in order to avoid ammonia volatilization, the phenol red concentration was increased, and the culture was not washed nor adjusted to an OD of 0.5 at 600 nm.
S. pasteurii DSM33 was cultivated overnight in shake flasks according to the pre-cultivation steps. To get a point of reference, 500 μL of the culture was diluted with 500 μL CaSo^{+} medium (black squares). In order to demonstrate the influence of an increased buffer capacity of the final sample, the culture sample was diluted with CaSo^{+} medium with extra MOPS buffer, resulting to a final concentration of 0.2 M MOPS, which can be considered to be a typical Good's buffer concentration in a culture medium. As stated in the main article, a higher buffer capacity of the sample resulted in a slower color change (blue dots) and therefore in an underdetermined urease activity. An increased sample OD600 was simulated by addition of CaSo^{+} medium with inactive yeast (X-SEED Cell-FX, Ohly, Hamburg). A higher OD600 results in an overall positive shift of the absorbance curve already at the starting point (red triangles), again having an impact on the determined urease activity. The hardly visible error bars depict the standard deviation.

SUPPLEMENTARY FILE 2 – SPECTRA OF DIFFERENT FLUORESCEIN

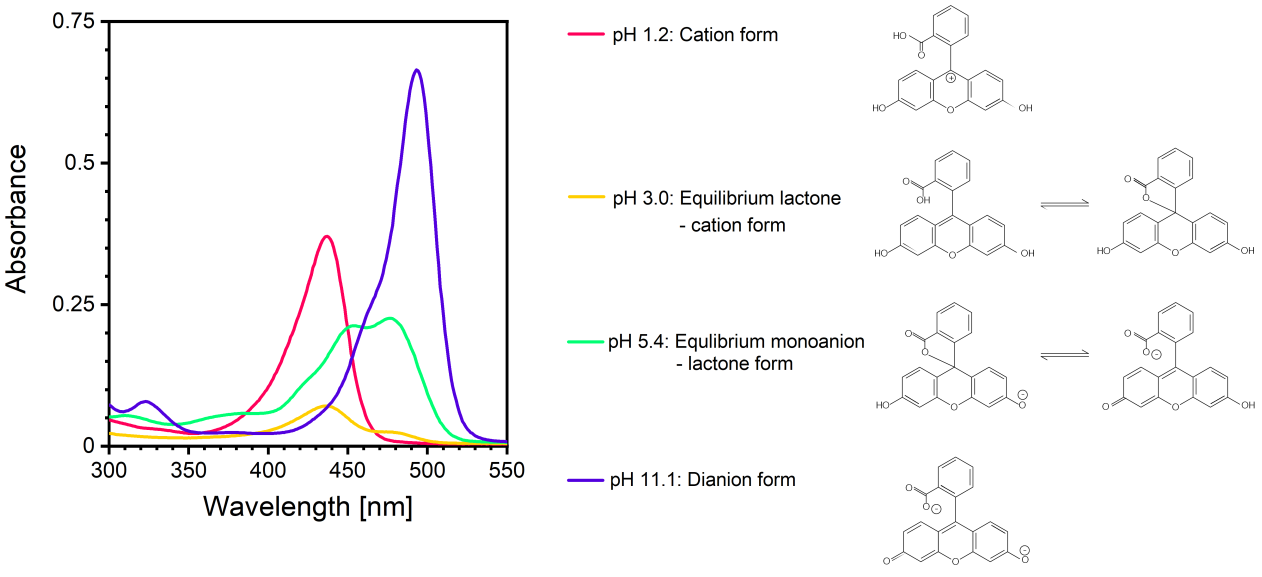

Figure S2. Adaptation of Figure 2, showing four representative spectra at different pH to illustrate the vastly different spectral properties of different fluorescein forms, based on Le Guern et al. (2020). For reagent preparation, NaOH is added to the concentrated stock solution. Consequently, mainly fluorescein in its dianion form is present in the solution. The absorbance of the stock solution at the isosbestic point is then measured and used to reliably prepare a dilution used for the final reagent.

SUPPLEMENTARY FILE 3 – DETERMINATION OF SAMPLE-TO-REAGENT RATIO, BUFFER SYSTEM AND BUFFER CONCENTRATION

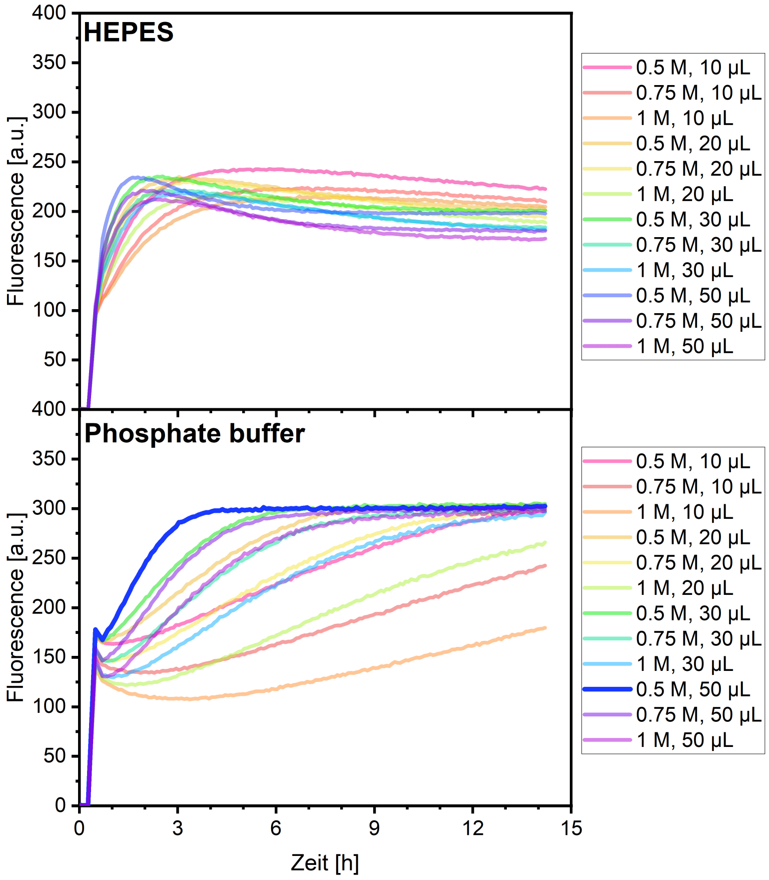

Figure S3. Emission signals of the full factorial optimisation experiment to find an applicable composition of the fluorescein reagent and a suitable sample-to-reagent ratio, using the same urease-positive microbial sample. The fluorescence is read using a BioLector I (Beckman Coulter Life Sciences, Baesweiler) (mean values, N = 2).

The original Stuart Broth consists of a phosphate buffer. However, the Good's buffer HEPES, having a similar pK_a value as a phosphate buffer, was also considered as an easy to prepare alternative instead of phosphate buffer for the fluorescein reagent. Therefore, both buffers (HEPES and phosphate) were tested, at buffer concentrations ranging from 0.5 to 1 M. Sample volumes from 10 to 50 μL were tested as well. Signals of reagent containing phosphate buffer were found to plateau more constantly than those of reagent containing HEPES. This was later explained by the quenching properties of HEPES (see Supplementary File S10). Therefore, phosphate buffer was chosen to be used for the fluorescein reagent. Buffer concentration and sample volume were found to both have opposite effects on the signal slope; a high buffer concentration slowed fluorescence increase, while higher sample volumes increased the fluorescence signal slope. These findings were expected, as more ureolytic sample results in faster urea hydrolysis, but increased buffer concentration results in a reduced pH shift. Finally, as higher sample volumes can be pipetted more reliably, a sample size of 50 μL (resulting in a sample-to-reagent ratio of 1:20) was found suitable. When adding 50 μL sample, the lowest buffer concentration of 0.5 M resulted in the fastest and most linear fluorescence signal (dark blue line). Based on these results, a buffer concentration even lower than tested here, namely 0.4 M, was found the most suiting for a reliable but fast assay procedure.

SUPPLEMENTARY FILE 4 – CORRELATION BETWEEN MICROPLATE READER AND BIOLECTOR I FLUORESCENCE MEASUREMENTS

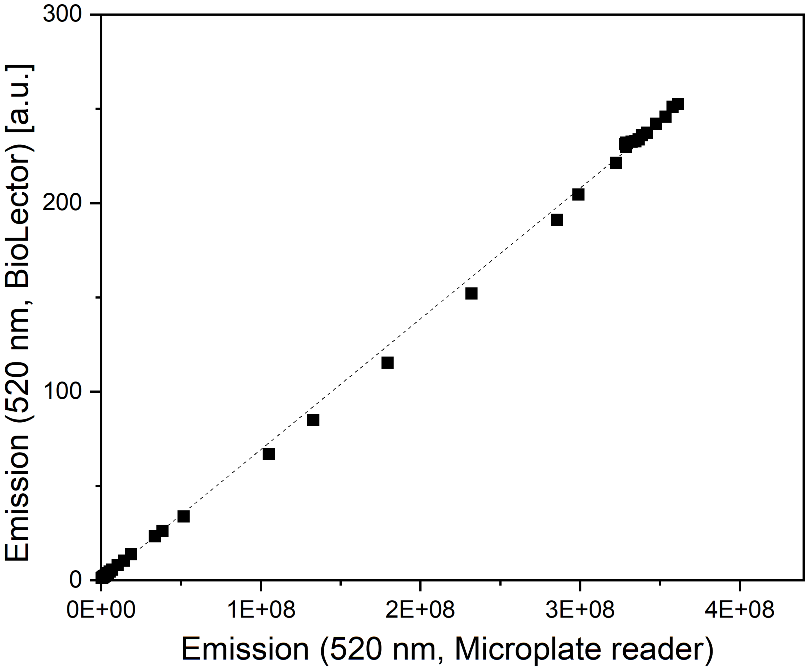

Figure S4. Graph illustrating the correlation (R^2 = 0.9991) of fluorescence measurements using the BioLector I (Beckman Coulter Life Sciences, Baesweiler) and a microplate reader (SpectraMax iD3, Molecular Devices, USA) (mean value, N = 2) at $\lambda_{Ex.}$ = 488 nm and $\lambda_{Em.}$ = 520 nm of a fluorescein solution at different pH. Data corresponding to Figure 2B of the main article.

SUPPLEMENTARY FILE 5 – INFLUENCE OF INITIAL REAGENT TEMPERATURE ON FLUORESCENCE SIGNAL

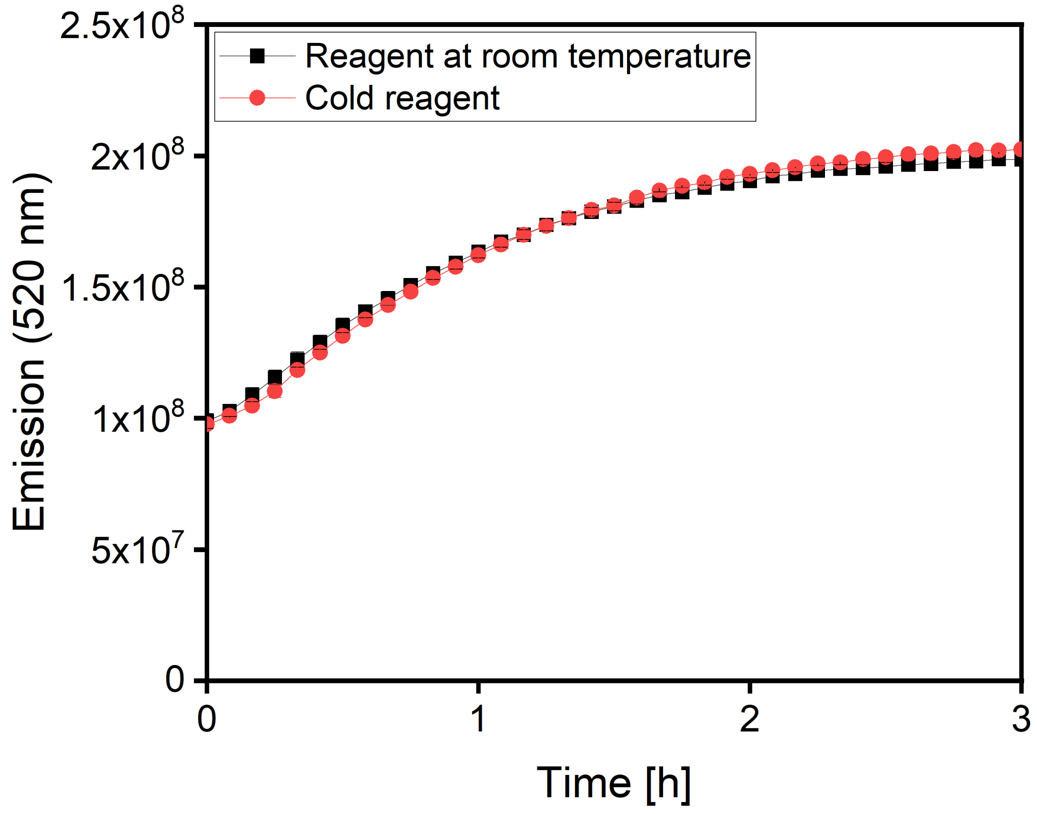

Figure S5. Emission signals of the same ureolytic sample measured with the identical fluorescein reagent, one half stored at room temperature, the other half stored in the laboratory fridge at 4 °C (N = 2). The fluorescence signals from samples with initially colder reagent are at the beginning slightly below the signals from the reagent stored at room temperature. The fluorescence is measured at 30 °C with a microplate reader (SpectraMax iD3, Molecular Devices, USA) as described in the manuscript. The error bars depict the standard deviation.

SUPPLEMENTARY FILE 6 – OD600 OF EXTRA TURBID SAMPLES

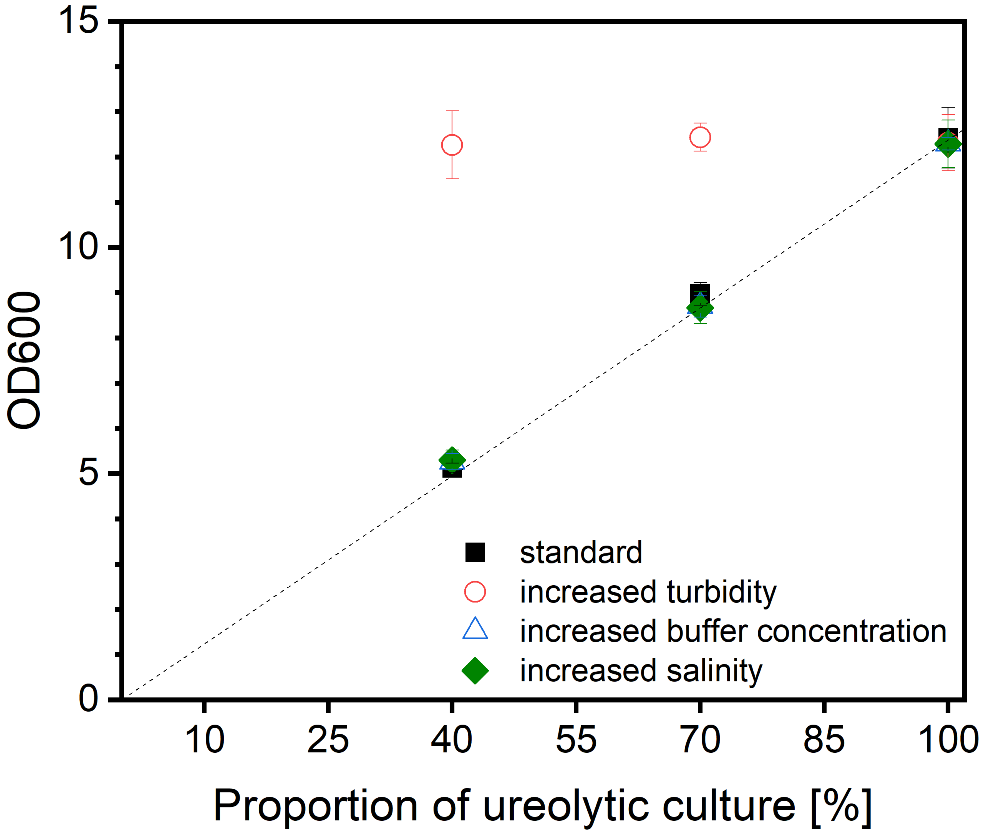

Figure S6. OD600 of samples prepared to quantify sample influences on assay accuracy (mean value, N = 3). In order to have a dilution sequence with the same OD600, inactive yeast (X-SEED Cell-FX, Ohly, Hamburg) was added to the samples, resulting in similar OD600 at every dilution step (red circles). As stated in the main article, an elevated sample buffer concentration was simulated by addition of MOPS (3-(N-morpholino)propanesulfonic acid) buffer to the sample (blue triangles). An increased sample salinity was simulated by the addition of extra NaCl to the sample (green triangles). The dotted line indicates a fit curve for the standard data (R^2 = 0.9999). The error bars depict the standard deviation.

SUPPLEMENTARY FILE 7 – 2D-FLUORESCENCE SPECTRA

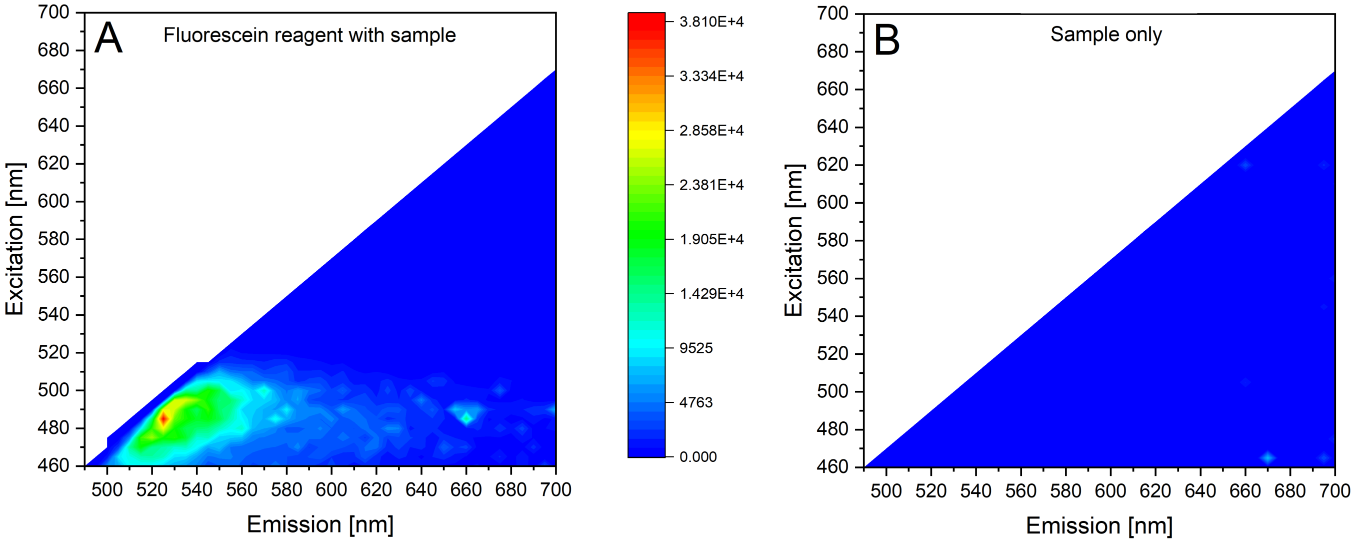

Figure S7. 2-D fluorescence spectra with excitation/emission wavelength pairs of **(A)** the fluorescein reagent after sample addition and **(B)** the same bacteria sample diluted with 0.9 % NaCl-solution instead of fluorescence reagent. As expected, the fluorescence signal of the reagent peaks at an excitation wavelength around 480 to 490 nm and an emission wavelength between 520 and 530 nm. Using the same scale, no relevant levels of biogenic green fluorescence of the bacterial sample can be detected. The same is true for measurements in the BioLector; the fluorescence of the culture itself never exceeded 1.1 a.u. during cultivation, which is negligible compared to the fluorescence signals resulting from the reagent ($>$ 280 a.u.). Please note, that the culture is also diluted 1:20 by addition to the reagent, further reducing any possible negative impact. Consequently, no direct interference from the biological matrix is expected.

SUPPLEMENTARY FILE 8 – UREASE ACTIVITY MEASURED VIA CONDUCTIVITY ASSAY

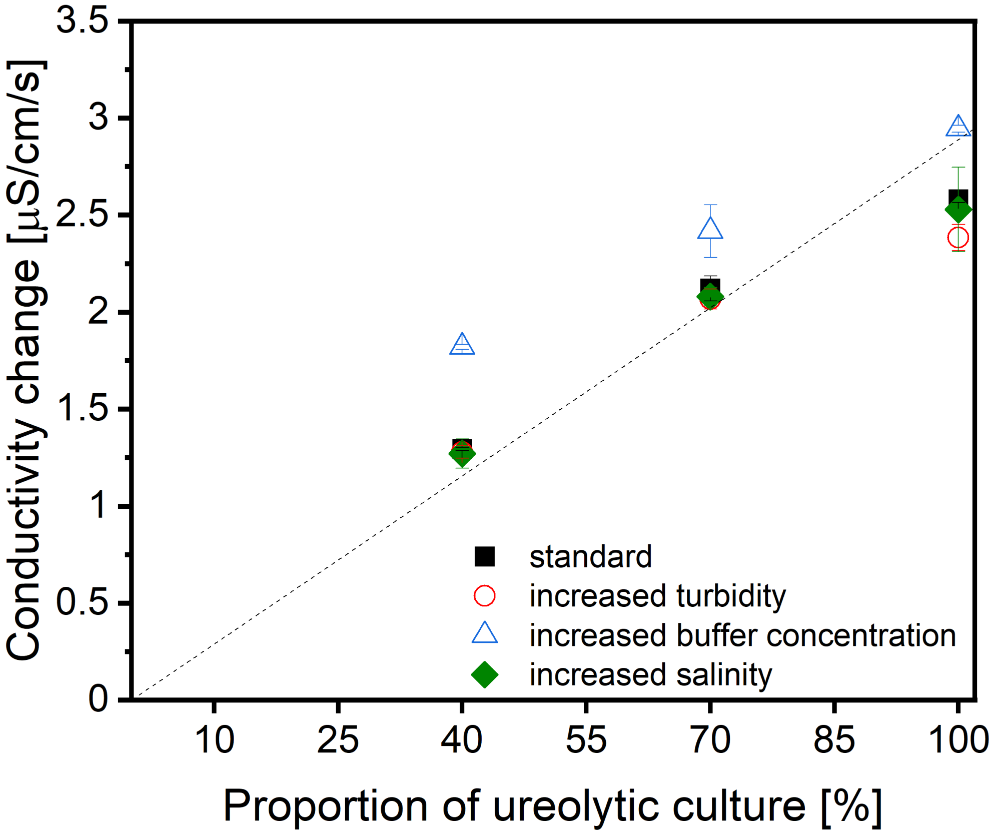

Figure S8. Conductivity change indicating urease activity of samples prepared to quantify sample influe-nces on assay accuracy (mean value, N = 3). Sample turbidity (red circles) and salinity (green triangles) do not have a notable impact on conductivity change. However, an increased buffer concentration (blue triangles) leads to higher urease activity (14 % to 41 %) compared to results from the standard sample (black rectangles), and therefore to a severe overestimation of the urease activity. The dotted line indicates a fit curve for the standard data (R^2 = 0.9876). The error bars depict the standard deviation.

SUPPLEMENTARY FILE 9 – ILLUSTRATION OF PHOTOBLEACHING EFFECT ON FLUORESCENCE MEASUREMENT

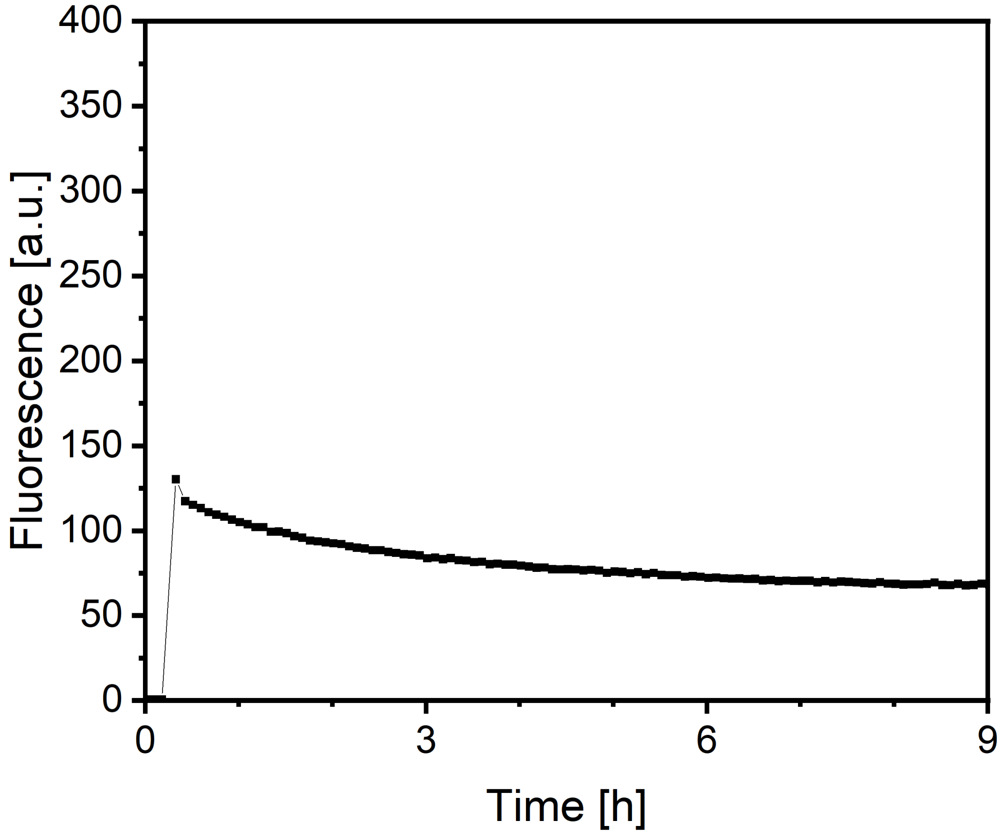

Figure S9. Emission signal of pure fluorescein reagent without any sample addition. The fluorescence signal decreases constantly, probably caused by photobleaching from fluorescence and backscatter measurements. The fluorescence is measured using the BioLector I (Beckman Coulter Life Sciences, Baesweiler).

SUPPLEMENTARY FILE 10 – STERN-VOLMER PLOTS

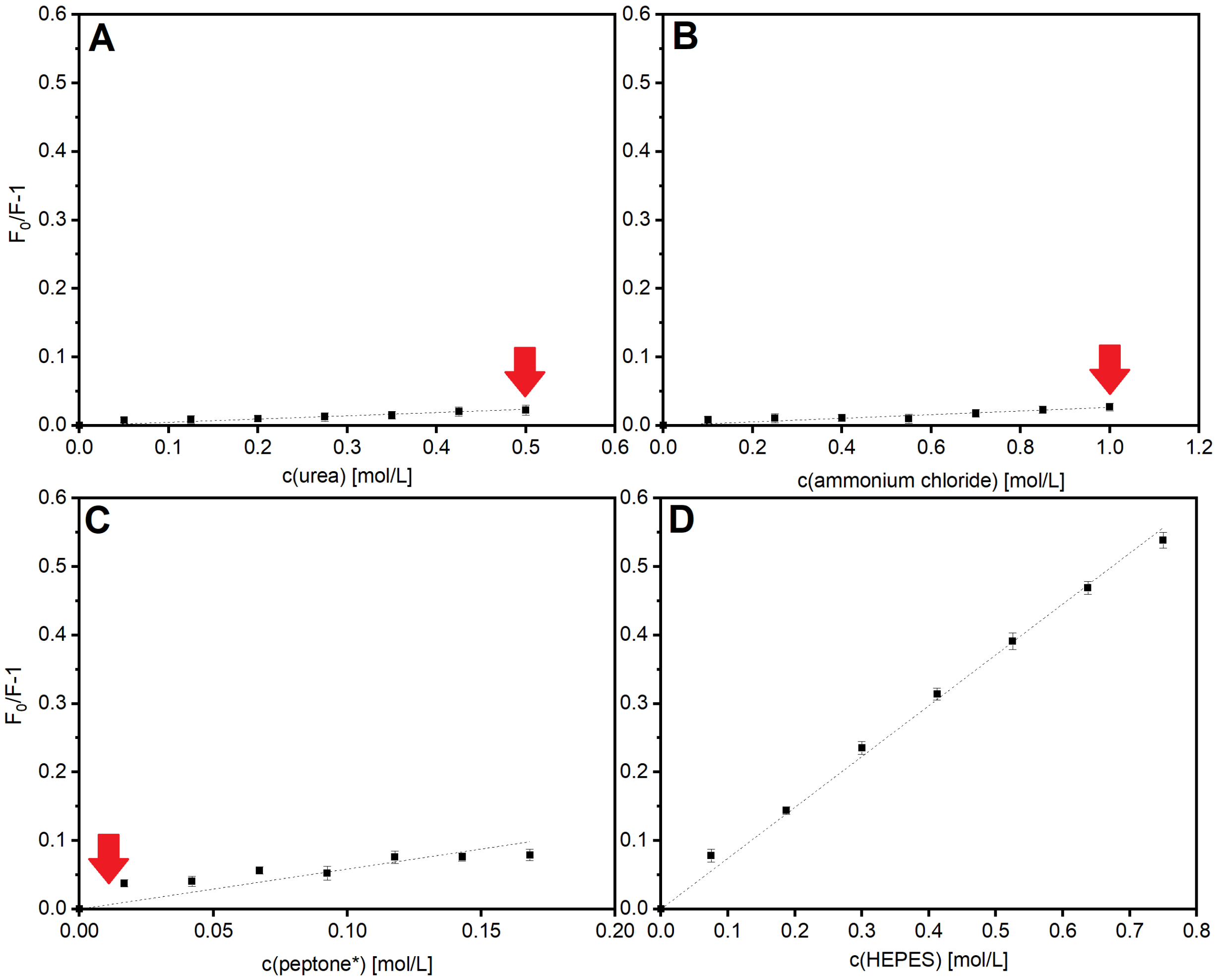

Figure S10. Stern-Volmer plots illustrating quenching properties of **(A)** urea, **(B)** ammonium chloride, **(C)** peptone and **(D)** HEPES. The y-axis corresponds to the quotient of the fluorescence emission without quenching substance to the fluorescence emission with a certain concentration of quenching substance minus one. In order to detect possible quenching properties of the fluorescein reagent component urea, a typical sample component peptone or the hydrolysis product ammonia, three Stern-Volmer plots were recorded using a microplate reader (SpectraMax iD3, Molecular Devices, USA). HEPES, which was also considered as possible buffer for the fluorescein reagent, was also tested. Fluorescence was measured at $\lambda_{Ex.}$ = 488 nm and $\lambda_{Em.}$ = 520 nm (mean value, N = 3). The pH was increased to over 10 for every sample, in order to eliminate effects from pH on fluorescence measurement. At concentrations expected during the assay (indicated by red arrows), neither urea, ammonium chloride or peptone strongly quench fluorescein and should therefore not notably impact the assay accuracy. However, HEPES strongly quenches fluorescein, thus could not be used as a buffer for the fluorescein reagent. The error bars depict the standard deviation.

SUPPLEMENTARY FILE 11 – DETERMINATION OF Z'-FACTOR AND STRICTLY STANDARDIZED MEAN DIFFERENCE

The Z'-Factor by Zhang *et al.* can be calculated with the following formula:

$$Z = 1 - \frac{(3\sigma_{pos} + 3\sigma_{neg})}{|\mu_{pos} - \mu_{neg}|} \tag{S1}$$

The Strictly Standardized Mean Difference can be calculated with this formula:

$$SSMD = \frac{\mu_{pos} - \mu_{neg}}{\sqrt{\frac{n_{pos}-1}{n_{pos}}\sigma_{pos}^2 + \frac{n_{neg}-1}{n_{neg}}\sigma_{neg}^2}} \tag{S2}$$

For both equations, μ indicate the mean value, σ the standard deviation and n the sample size. The indices pos and neg indicate if the values are calculated from the positive or the negative control samples, respectively. All parameters are without units.

As stated in the main article, a microplate reader measurement was performed to gather data for the assay evaluation. The resulting values are listed here:

$\mu_{pos} = 235,626,670$

$\sigma_{pos} = 5,671,200$

$n_{pos} = 4$

$\mu_{neg} = 99,710,359$

$\sigma_{neg} = 2,889,649$

$n_{neg} = 4$

Based on these values, the Z'-Factor was found to be 0.94 and the SSMD was found to be 24.7. Both evaluation factors rank the assay performance as "excellent", as seen in Table S1.

Table S1. Criteria to evaluate screening assay quality (Zhang *et al.*, 1999; Buß *et al.*, 2016)

Z'-factor	SSMD	Performance
1 ≥ Z' ≥ 0.5	SSMD ≥ 3.0	Excellent assay
0.5 > Z' ≥ 0		Weak assay
> 0	SSMD ≤ 3.0	Screening impossible

SUPPLEMENTARY FILE 12 – COST COMPARISON OF UREASE ACTIVITY ASSAYS

For cost calculations, only costs for reagent components and microplates or cuvettes are considered. Instrument acquisition costs (e.g. for microplate reader, conductivity probe), costs for other consumables (e.g. pipette tips), labour costs or costs for disposal are not included in the cost calculation presented here. Possible cost optimisation for any of these assays is also not taken into account. All net costs for reagent components, microplates or cuvettes are taken from www.carlroth.com (27.01.2022).

Fluometric assay

Table S2. Reagent cost calculation for the assay presented in this study

Reagent component	Net costs	Relative costs	Required amount	Costs for reagent (1 L)
Fluorescein	110.9 Euro/500g	0.22 Euro/g	0.1 g/1000mL	0.02 Euro
Urea	239.5 Euro/25000g	0.01 Euro/g	30 g/1000mL	0.29 Euro
K_2HPO_4	37.5 Euro/1000g	0.04 Euro/g	5.30 g/1000mL	0.20 Euro
KH_2PO_4	485 Euro/25000g	0.02 Euro/g	51.27 g/1000mL	0.99 Euro
			Sum:	1.50 Euro

1 L reagent results in 1052 urease activity measurements. Therefore, one single measurement costs less than 0.01 Euro. One 24 multiwell plate allows for 23 urease activity measurements (one well reserved as blank). As one 24 multiwell plate costs 2.06 Euro, measuring 23 samples results in total costs of 2.10 Euro.

Conductivity assay

Table S3. Reagent cost calculation for the conductivity assay adapted from Whiffin (2004)

Reagent component	Net costs	Relative costs	Required amount	Costs for reagent (1 L)
Urea	239.5 Euro/25000g	0.01 Euro/g	66 g/1000mL	0.63 Euro
			Sum:	0.63 Euro

1 L reagent results in 41 urease activity measurements. For 23 samples, 552 mL urea solution and no consumables are required, resulting in total costs of 0.35 Euro for this number of samples.

Colorimetric assay

Table S4. Reagent cost calculation for the Berthelot assay, consisting of two reagents labelled with (1) and (2)

Reagent component	Net costs	Relative costs	Required amount	Costs for reagents (100 mL + 200 mL)
Phenol (1)	68.9 Euro/500g	0.14 Euro/g	7 g/100mL	0.96 Euro
Sodium nitroprusside (1)	319.0 Euro/500g	0.64 Euro/g	0.034 g/100mL	0.02 Euro
NaOH (2)	15.9 Euro/1000g	0.02 Euro/g	2.96 g/200mL	0.05 Euro
$Na_2HPO_4 \cdot 7H_2O$ (2)	23.9 Euro/500g	0.05 Euro/g	22.29 g/200mL	1.07 Euro
NaClO (12 % v/v) (2)	38.9 Euro/10000mL	0.004 Euro/g	16.6 mL/200mL	0.06 Euro
			Sum:	2.16 Euro

Both reagents allow for 500 urease activity measurements. The colorimetric assay is performed using a spectrophotometer, requiring in total 48 cuvettes for 23 samples including blanks. Every sample requires two measurements, one before and one after ureolytic activity, in order to measure total ammonia increase. One cuvette costs 0.30 Euro, resulting in total costs of 14.12 Euro for 23 samples.
